# Advances in Genetic Engineering Technology and Its Application in the Industrial Fungus *Aspergillus oryzae*

**DOI:** 10.3389/fmicb.2021.644404

**Published:** 2021-02-23

**Authors:** Feng-Jie Jin, Shuang Hu, Bao-Teng Wang, Long Jin

**Affiliations:** Co-Innovation Center for Sustainable Forestry in Southern China, College of Biology and the Environment, Nanjing Forestry University, Nanjing, China

**Keywords:** Aspergillus oryzae, fermentation industry, genetic engineering technology, cell factory, heterologous protein production

## Abstract

The filamentous fungus *Aspergillus oryzae* is an important strain in the traditional fermentation and food processing industries and is often used in the production of soy sauce, soybean paste, and liquor-making. In addition, *A. oryzae* has a strong capacity to secrete large amounts of hydrolytic enzymes; therefore, it has also been used in the enzyme industry as a cell factory for the production of numerous native and heterologous enzymes. However, the production and secretion of foreign proteins by *A. oryzae* are often limited by numerous bottlenecks that occur during transcription, translation, protein folding, translocation, degradation, transport, secretion, etc. The existence of these problems makes it difficult to achieve the desired target in the production of foreign proteins by *A. oryzae*. In recent years, with the decipherment of the whole genome sequence, basic research and genetic engineering technologies related to the production and utilization of *A. oryzae* have been well developed, such as the improvement of homologous recombination efficiency, application of selectable marker genes, development of large chromosome deletion technology, utilization of hyphal fusion techniques, and application of CRISPR/Cas9 genome editing systems. The development and establishment of these genetic engineering technologies provided a great deal of technical support for the industrial production and application of *A. oryzae*. This paper reviews the advances in basic research and genetic engineering technologies of the fermentation strain *A. oryzae* mentioned above to open up more effective ways and research space for the breeding of *A. oryzae* production strains in the future.

## Introduction

*Aspergillus oryzae* is an important filamentous fungus that is widely applied in the traditional fermentation and food processing industries, such as soy sauce, soybean paste, and sake brewing. This long history of its widespread use in the food industry has led to *A. oryzae* being recognized by the Food and Drug Administration (FDA) of the United States as Generally Recognized as Safe (GRAS) organisms. Its safety is also supported by the World Health Organization (FAO/WHO) (Kobayashi et al., 2007).

In addition, *A. oryzae* has a strong capacity to secrete large amounts of hydrolytic enzymes (such as amylases and proteases); therefore, it has also been used in the enzyme industry as a cell factory for the production of numerous native and heterologous enzymes. In addition to protein secretion ability, *A. oryzae* also has a strong synthesis ability, rapid growth, and easy culture, among its advantages. Compared to *Escherichia coli* and yeast *Saccharomyces cerevisiae*, *A. oryzae* has a strong posttranslational modification function; therefore, it is widely used to produce a variety of enzyme preparations (Lissau et al., 1998; Hama et al., 2008; Merz et al., 2015) and in recent years has also been used in the production of secondary metabolites (Yamada et al., 2014; Kan et al., 2019; Liu et al., 2019; Nagamine et al., 2019; Morishita et al., 2020; Oikawa, 2020a).

In the past few decades, research on *A. oryzae* has focused on breeding techniques and developing methods for brewing. Despite its considerable commercial importance, the study of fundamental biological processes in *A. oryzae* was delayed in comparison with the yeast *S. cerevisiae*. The main reasons are that *A. oryzae* is an imperfect fungus that does not have a sexual life cycle and forms multinucleate conidia, making classical genetic manipulation difficult to implement (Kitamoto, 2015). Therefore, it is difficult to study this organism using conventional genetic methods. The production and secretion of foreign proteins by *A. oryzae* are often limited by numerous bottlenecks during transcription, translation, protein folding, translocation, degradation, transport, secretion, etc. (Conesa et al., 2001; Ohno et al., 2011). The existence of these problems makes it difficult to achieve the desired target in heterologous protein production by *A. oryzae*.

In 2005, the genome sequence of *A. oryzae* RIB40 was deciphered (Machida et al., 2005), which marked the advent of the whole genome era of *A. oryzae*, and the combination of genetic engineering technology further promoted the development of *A. oryzae*. Subsequently, many other *A. oryzae* strains were sequenced, such as 3.042, which is extensively used for the production of both soy sauce and other fermented foods in China (Zhao et al., 2012). Comparative genomics analysis showed some strain-specific genes that encode putative proteins potentially involved in cell growth, salt tolerance, environmental resistance and flavor formation (Zhao et al., 2013). The 37-Mb genome of *A. oryzae* RIB40 was predicted to contain more than 12,000 genes, which is 7–9 MB larger than the genomes of *Aspergillus nidulans* and *Aspergillus fumigatus* (Galagan et al., 2005; Machida et al., 2005; Nierman et al., 2005). The completed genomic studies using expressed sequence tag analyses (Machida, 2002; Akao et al., 2007) combined with the development of genetic engineering technology have made it possible to overcome these deficiencies and promoted the study of heterologous protein production in *A. oryzae* and the breeding of new strains for industrial production (Vongsangnak et al., 2008; Zhu et al., 2013). Regarding genetic engineering to solve the bottleneck problems in heterologous protein production in *A. oryzae*, this article elaborated on several aspects, such as optimization of the genetic transformation system and the improvement of transformation efficiency, construction of protease gene-deleted strains, development of fusion expression strategies, optimization of exogenous gene expression systems, and development of other genetic engineering techniques to improve the production of foreign proteins. These advances in basic research and genetic engineering technology have greatly improved the production and application of *A. oryzae*.

## Development of Transformation Systems of *A. oryzae*

In industrial applications, the establishment of a highly efficient genetic transformation system is a prerequisite for genetic manipulation and recombinant gene expression in *A. oryzae*. Compared to *E. coli* and yeast *S. cerevisiae*, the *A. oryzae* cell wall is hard and not easily broken, which is an obstacle for gene introduction into host cells in later genetic transformation experiments. Therefore, some rapid and convenient transformation methods, such as the electroporation and gene gun methods, are not suitable for *A. oryzae* genetic transformation due to their extremely low transformation efficiency. By contrast, another conventional genetic transformation method, which is a polyethylene glycol (PEG)/CaCl_2_-mediated protoplast transformation system, was widely utilized in *A. oryzae*.

### Development and Application of Selective Marker Genes

The application of recombinant DNA methodology in *A. oryzae* allows advanced molecular genetic analyses, which makes it possible to introduce DNA fragments into the host to produce heterologous proteins (Kitamoto, 2002). For effective molecular genetic analysis and protein production, suitable and stable hosts are needed as genetically engineered strains to transform the selected target genes. In previous research, several auxotrophic marker genes, such as *pyrG*, *argB*, *niaD*, and *sC* (Gomi et al., 1987; Mattern et al., 1987; Unkles et al., 1989; Yamada et al., 1997), and dominant selectable markers, such as *amdS* and *ptrA* (Gomi et al., 1991; Kubodera et al., 2000), have been developed and used in the genetic transformation of *A. oryzae*. Recently, a new pyrithiamine resistance marker gene, *thiI*, was also identified for use in genome editing by selecting mutants resistant to pyrithiamine from UV-mutagenized *A. oryzae* strains (Todokoro et al., 2020). In addition to these, a bleomycin resistance marker gene (*blmB*) and a carboxin resistance marker gene [*AosdhB*(cxr)] also were developed for *A. oryzae* transformation (Shima et al., 2009; Suzuki et al., 2009). However, all these transformation techniques only use a single gene as a selective marker gene; therefore, several multitransformation systems of *A. oryzae* containing multiple selective marker gene-defective strains were constructed, which could carry out gene engineering operations such as introduction or deletion of multiple genes in the same host. For this purpose, Yamada et al. (1997) further developed a double auxotrophic transformation system (*niaD*^–^, *sC*^–^) that can conduct two genetic manipulations in a single host strain. On this basis, triple auxotrophic mutants (*niaD*^–^, *sC*^–^, *adeA*^–^/*adeB*^–^) were further obtained from *A. oryzae* by UV mutagenesis, which added a new adenine auxotrophy (Jin et al., 2004a). In addition, a quadruple auxotrophic host transformation system was further developed by *argB* gene deletion combined with exploiting adenine auxotrophy (Jin et al., 2004b). Among these selection marker genes, the *pyrG* gene, which encodes an orotidine-5′-phosphate (OMP) decarboxylase, has been used for marker recycling that allows multiple genetic manipulations in the *Aspergillus* species (Nielsen et al., 2006; Maruyama and Kitamoto, 2008) because *pyrG*-deficient strains can be positively selected by 5-fluoroorotic acid (5-FOA), which is converted to a toxic compound, to strains expressing *pyrG*. These selectable marker genes mentioned above are summarized in Table 1. In addition, the Cre-*loxP* site-specific recombination system, which has been widely applied in a number of organisms, such as bacterial, yeast, plant, and mammalian cells (Sauer, 1987; Gu et al., 1993; Sieburth et al., 1998), was also developed for multiple gene integrations and deletions in *A. oryzae* (Mizutani et al., 2012; Zhang et al., 2017). This system is used for site-specific recombination between two *loxP* sites mediated by a Cre recombination protein derived from bacteriophage P1 (Abremski et al., 1986).

**TABLE 1:** Selectable marker genes used in *Aspergillus oryzae.*

Selectable marker gene	Description	References
*niaD*	The *niaD* gene encodes a nitrate reductase, and the *niaD*^–^ mutants display chlorate resistance	Unkles et al., 1989
*pyrG*	The *pyrG* gene encodes an orotidine-5′-phosphate (OMP) decarboxylase, and *pyrG*-deficient strains can be positively selected by 5-fluoroorotic acid (5-FOA)	Mattern et al., 1987
*argB*	The *argB* gene encodes an ornithine carbamoyltransferase and allows the complementation of the arginine auxotrophic mutation	Gomi et al., 1987
*sC*	*sC* marker gene encodes an ATP sulfurylase, and the *sC*^–^ mutants can be isolated by positive selection for selenate resistance	Yamada et al., 1997
*adeA*	The *adeA* gene encodes a phosphoribosylaminoimidazolesuccinocarboxamide synthase of purine biosynthetic pathway and could complement the adenine auxotrophic mutants	Jin et al., 2004a
*adeB*	The *adeB* gene encodes a phosphoribosylaminoimidazole carboxylase of purine biosynthetic pathway and could complement the adenine auxotrophic mutants	Jin et al., 2004a
*amdS*	The *amdS* gene encodes an acetamidase that is required for acetamide utilization	Gomi et al., 1991
*ptrA*	The *ptrA* is a pyrithiamine-resistant gene that was cloned from a pyrithiamine resistant mutant of *A. oryzae*	Kubodera et al., 2000
*thiI*	The *thiI* gene encodes a thiamine transporter gene, and its mutant showed pyrithiamine resistance	Todokoro et al., 2020
*blmB*	The *blmB* gene encodes bleomycin *N*-acetyltransferase from bleomycin-producing *Streptomyces verticillus* and is responsible for the bleomycin resistance	Suzuki et al., 2009
*AosdhB*(cxr)	*AosdhB* encodes a succinate dehydrogenase gene, and its mutation [*AosdhB*(cxr)] confers the carboxin resistance.	Shima et al., 2009

### Chromosomal Engineering Technology

One of the major reasons for the lag in basic research on *A. oryzae* is the low efficiency of homologous recombination transformation, which makes it difficult to obtain gene deletion strains. Takahashi et al. (2006) significantly improved the homologous recombination efficiency of *A. oryzae* by deleting non-homologous end-joining (NHEJ)-related genes such as *ku70* and *ku80*, thus enhancing the gene deletion efficiency of *A. oryzae*. Next, some studies also showed that the disruption of another NHEJ-related *ligD* gene significantly improved gene-targeting efficiency in *A. oryzae* (Maruyama and Kitamoto, 2008; Mizutani et al., 2008). With the significant improvement in the efficiency of homologous recombination, deletion technology of large chromosomal segments, including loop-out recombination and replacement-type recombination methods, has been developed (Takahashi et al., 2008, 2009), providing technical support for the further deletion of unnecessary and even harmful large chromosomal segments from the chromosomes in *A. oryzae*. Although *A. oryzae* is regarded as an industrially important fungus, its genome sequence still contains several gene clusters related to the synthesis of toxins, such as imperfect clustered genes related to aflatoxin biosynthesis and cyclopiazonic acid production. Although these toxic secondary metabolites are not normally synthesized, they are also at a certain risk, and these gene clusters can be deleted through the deletion technology of large chromosomal segments. On this basis, Jin et al. (2010) conducted a chromosome-shortening experiment, and most of the unnecessarily long DNA fragments and potential toxin synthesis-related gene clusters on chromosome 7 of *A. oryzae* were successfully removed, which not only promoted the production of amylase but also avoided the production of some byproducts that are possibly harmful to human health. By eliminating non-essential chromosomal segments and retaining only the genes that maintain basic physiological functions, we will gain a deeper understanding of the physiological characteristics of this organism and the essential gene functions for its industrial use. The development of these techniques pave a new way for the future production and utilization of *A. oryzae*.

In addition, *A. oryzae* was thought to undergo asexual reproduction, and no sexual stage has been reported; therefore, it has certain limitations in production and application. For instance, breeding better production strains cannot be achieved by sexual hybridization. To solve this problem, protoplast fusion technology was developed, and a further reduced chromosome *A. oryzae* mutant was constructed with a shortened chromosomes 7 and 8, both of which include a large number of non-syntenic blocks by using this protoplast fusion technique (Hara et al., 2012). The large chromosome segment deletion technique combined with the protoplast fusion technique has effectively solved the problem that has puzzled *A. oryzae* researchers for many years and provided a good technical means for better chromosome processing in the future to obtain the breeding of excellent *A. oryzae* production strains. In addition, using the large-scale chromosome deletion technique, a member of the basic helix-loop-helix (bHLH) transcription factor family, SclR, was identified and characterized (Jin et al., 2009, 2011b). Studies have shown that SclR cannot only promote sclerotia formation by promoting hyphal fusion but also effectively promote nucleus fusion between different strains (Wada et al., 2014), which provides a more feasible space for breeding excellent *A. oryzae* strains in the future. These studies indicated that the deletion technique of large chromosome segments is also of great value for the discovery and exploration of unknown functional genes.

### *Agrobacterium*-Mediated Transformation System

*Aspergillus oryzae* is resistant to most common antifungal antibiotics that are often used as selective agents for transformation experiments of filamentous fungi (Suzuki et al., 2009); therefore, *A. oryzae* transformation systems were developed mainly based on nutritional selectable markers for complementation of auxotrophic strains (Gomi et al., 1987; Mattern et al., 1987). Currently, PEG-mediated protoplast transformation is the common method utilized for fungal transformation experiments (Liu and Friesen, 2012). The *Agrobacterium tumefaciens*-mediated transformation (ATMT) system is another method that was developed and used for genetic transformation in the *Aspergilli* species (Michielse et al., 2005). The approach usually requires a binary vector carrying T-DNA (transfer DNA), an *A. tumefaciens* strain that harbors a helper plasmid with *vir* (virulence) supporting the T-DNA transfer process, and a suitable recipient (Michielse et al., 2005; Frandsen, 2011). Using the ATMT method to transfer DNA into the fungal genome has been shown to be more efficient and simpler to implement in different filamentous fungi, while PEG-mediated protoplast transformation is more laborious and time-consuming for protoplast preparation (Idnurm et al., 2017; Weyda et al., 2017). Recently, the efficient approach was used to successfully construct uridine/uracil auxotrophic mutants by deleting the *pyrG* gene in different industrial fungus *A. oryzae* wild-type strains, and the genetic transformation efficiency by *A. tumefaciens* was higher and reached approximately 0.1% using the *pyrG* selectable marker and these auxotrophic mutants (Nguyen et al., 2016, 2017). These generated auxotrophic mutants were also used to successfully express the DsRed fluorescent reporter gene under the control of the *A. oryzae* alpha-amylase gene (*amyB*) promoter by the ATMT method. Furthermore, using the ATMT method, a useful dual selection marker system was developed for genetic manipulations in an industrial *A. oryzae* 3.042 strain by constructing a uridine/uracil auxotrophic mutant and pyrithiamine (PT) genes as selection markers (Sun et al., 2019). In summary, this ATMT method can simplify transformation procedures and improve the efficiency of genetic manipulations. This strategy can be applied in functional gene research and the production of recombinant proteins by the industrial fungus *Aspergillus* strains through genetic transformation systems based on nutrition markers.

### CRISPR/Cas9-Based Genome Engineering

The increase in the number of whole genomes available for fungal species provided a wider range of possibilities for gene manipulation in filamentous fungi. In recent years, a versatile genomic editing technique, the CRISPR (clustered regularly interspaced short palindromic repeat)–Cas9 (CRISPR-related nuclease 9) system, has been rapidly developed and widely applied in filamentous fungi. The advance of this technology has revolutionized biological research and led to innovative applications in a wide range of fields, showing great prospects in the research and application of filamentous fungi (Song et al., 2019).

The CRISPR/Cas9 system is divided into two categories, including six types and 19 subtypes (Shmakov et al., 2017). Among them, the type II system is a simpler CRISPR system than other systems that have been widely used. The type II CRISPR/Cas9 system contains Cas9 (nuclease), mature crRNA (CRISPR associate RNA), tracrRNA (*trans*-activating crRNA), and RNaseIII. The crRNA and tracrRNA have been modified and linked into a single-guide RNA (sgRNA) (Jinek et al., 2012) that could efficiently guide the Cas9 nuclease to the target sequences to cut the DNA. Currently, CRISPR/Cas9 technology has been widely used for genome editing in yeasts/fungi, plants, mammalian cells, and others (Cong et al., 2013; van Erp et al., 2015; Schiml and Puchta, 2016). In fungi, the CRISPR/Cas9 genome editing system was first introduced into *S. cerevisiae* (DiCarlo et al., 2013) and then further applied to *Trichoderma reesei*, *Neurospora crassa*, and *Aspergillus nidulans* (Liu et al., 2015; Matsu-Ura et al., 2015; Nodvig et al., 2018). In addition, the CRISPR/Cas9 system was discovered in bacteria or archaea, and therefore, the Cas9-encoding gene usually needs to be fungal codon-optimized and a nuclear localization signal when the genome editing system is used in fungi (Generoso et al., 2016; Nodvig et al., 2018). In recent years, some studies have shown that the CRISPR/Cas9 technique has been used in the production of recombinant proteins in the *Aspergilli* species (Dong et al., 2020; Rojas-Sanchez et al., 2020).

To develop the CRISPR/Cas9 genome editing technique in *A. oryzae*, plasmids that express the Cas9 nuclease gene and sgRNAs were constructed and introduced into an *A. oryzae* strain for the mutagenesis of target genes (Katayama et al., 2016). In this study, the codon usage of *cas9* with a sequence encoding an SV40 nuclear localization signal at its 5′- and 3′-ends was optimized to improve the nuclear localization of Cas9 nuclease. The generated transformants contained mutations in which each target gene showed the expected phenotype. Mutation rates between 10 and 20% with 1 bp of deletion or insertion are the most common induced mutations, and genome editing technology will contribute to the efficient use of targeted mutagenesis in many *A. oryzae* industrial strains without a clear genetic background. To further improve the targeting efficiency in *A. oryzae* industrial strains, the *ligD* gene involved in NHEJ was mutated by employing the CRISPR/Cas9 system (Nakamura et al., 2017). The gene targeting efficiency of the generated *ligD* mutant was examined using the *ecdR* gene, which encodes a regulator involved in the early stage of conidiophore development in the *A. oryzae* (Jin et al., 2011a). The results indicated that the deletion efficiency was significantly improved and reached approximately 60∼80% in the *ligD* mutants.

In addition to food fermentation and industrial production of recombinant proteins, *A. oryzae* has also been used for heterologous production of useful secondary metabolites; therefore, multiple steps of genetic manipulation are required.

An improved CRISPR/Cas9 approach that includes an *AMA1*-based autonomously replicating plasmid harboring the pyrithiamine resistance marker gene *ptrA* was further developed (Katayama et al., 2019). The conditional expression of the *Aoace2* gene in the *AMA1*-based plasmid significantly repressed the growth of fungi, which made it possible for forced plasmid recycling and allowed repeated genome editing. This study showed that the repeatable marker-free genome editing approach can effectively delete or integrate multiple genes in the industrial *A. oryzae* strain (Katayama et al., 2019). In the production experiment of human antibody (anti-TNFα antibody adalimumab) by *A. oryzae*, the CRISPR/Cas9 technique was used for the deletion of the *Aooch1* gene, which encodes a key enzyme for the hypermannosylation process, to detect the binding ability of the recombinant antibody with FcγRIIIa (Huynh et al., 2020). In addition, the CRISPR/Cas9 system was also used to delete *AoGld3*, which is one of 45 glycerol dehydrogenase genes in *A. oryzae*, to investigate its effect on the production of the secondary metabolite kojic acid. The result showed that the deletion of *AoGld3* resulted in the inhibition of kojic acid production by affecting the expression of *kojA* and *kojR*, which encode an enzyme and a transcription factor involved in the kojic acid biosynthesis (Fan et al., 2020). In addition, a platinum-fungal TALEN (Transcription activator-like effector nucleases) -based genome editing technique has also been developed for targeted gene mutations in *A. oryzae* (Mizutani et al., 2017). The platinum-fungal TALEN technique resulted in various different mutation patterns with almost half of deletions larger than 1 kb in the target region. Taken together, these genome editing technologies will greatly enhance the convenience of genetic manipulation in a wide range of industrial production strains and significantly contribute to efficient molecular breeding of these industrial strains.

## Study on Heterologous Gene Expression and Protein Secretion Systems

*Aspergillus oryzae* has attracted increasing attention due to its wide use as a cell factory in the study of exogenous gene expression and heterologous protein production in recent years. An understanding of how optimize the exogenous gene expression system is of great significance to the industrial application of *A. oryzae*. By analyzing the advantages and disadvantages of the exogenous gene expression system of *A. oryzae* and combining genetic engineering technology developed in recent years, the exogenous gene expression of *A. oryzae* can be improved by making full use of the advantages while avoiding the disadvantages.

### Disruption of Proteinase Genes in *A. oryzae*

Compared with the production and secretion system of prokaryotes such as *E. coli*, exogenous genes expressed in *A. oryzae* undergo eukaryotic posttranslational modifications, including glycosylation and protein folding. Therefore, *A. oryzae* is considered as one of the most advantageous hosts for expressing recombinant proteins from higher eukaryotes (Fleissner and Dersch, 2010). The production of heterologous proteins is limited by many factors, such as the transcriptional level, translation level, secretory process, and extracellular degradation (Jeenes et al., 1991). Therefore, several bottleneck genes related to proteases, secretion pathways, cell polarity establishment, and metabolism must be manipulated to breed favorable hosts for heterologous protein production.

Among them, the strong protease activity of *A. oryzae* is the most important reason to inhibit the production of heterologous proteins because heterologous proteins are more easily broken down by proteases than the organism’s own proteins. To improve the yield of heterologous proteins, researchers have tried various biological methods. For instance, downstream processing at low temperature, early separation of products from proteases, application of protease inhibitors, etc. can reduce hydrolysis; however, the effect is not optimistic because most heterologous proteins could be degraded in the process of protein production (Jin et al., 2007). Using a quadruple auxotrophic host (Jin et al., 2004b), Jin et al. (2007) expressed the heterologous human lysozyme (HLY) gene by disrupting an intracellular acid protease PepE and a tripeptidyl peptidase TppA genes, and the double disruption led to an increase (63%) in HLY production in comparison to the control. In this study, the *HLY* gene fused with an *A. oryzae* α-amylase (AmyB) gene was expressed, the Kex2 cleavage site was inserted between them, and HLY was successfully processed and dissociated from the native AmyB protein and secreted into the medium. In addition, a systematic deletion analysis of five proteinase genes (*pepA*, *pepE*, *tppA*, *alpA*, and *palB*) in the HLY-producing strain was performed, and comparative analysis indicated that deletion of the *tppA* gene resulted in the highest increase (36%) in lysozyme activity of the HLY-producing strain (Jin et al., 2007). On this basis, a quintuple protease gene disrupted strain (*tppA*, *pepE*, *dppIV*, *dppV*, and *nptB*) (Yoon et al., 2009) and an additional ten protease gene disrupted strain with five additional deleted genes (*alpA*, *pepA*, *AopepAa*, *AopepAd*, and *cpI*) (Yoon et al., 2011) were successively constructed using *pyrG* marker recycling combined with a highly efficient gene targeting background (△*ligD*). The results showed that the yield of HLY and bovine chymosin (CHY) protein in the ten protease gene disruptants was significantly enhanced, which was 3.2 and 3.8 times that of the control strains, respectively.

To further solve the bottlenecks limiting the production of heterologous protein by *A. oryzae*, another tripeptidyl peptidase gene, *AosedD*, which may be involved in the proteolytic degradation of recombinant proteins, was selected to construct its deletion strain for the production of heterologous proteins. In the *AosedD* deletion mutant, the production levels of recombinant HLY and CHY improved approximately 1.7- and 2.9-fold, respectively, relative to their control strains (Zhu et al., 2012). The results show that AoSedD is one of the main proteases involved in the degradation of heterologous recombinant proteins in *A. oryzae*.

### Optimization of Heterologous Protein Secretion Pathways

Another important bottleneck in the production of heterologous proteins in filamentous fungi is the secretion of the proteins (Conesa et al., 2001). The unfolded protein response (UPR) is an intracellular regulatory pathway that activates the genes involved in folding, quality control, transport, and other functions of secreted proteins. The overexpression of heterologous proteins may result in the overload of unfolded proteins in the endoplasmic reticulum (ER) (Ron and Walter, 2007). The response is accompanied by the transcription activation of ER protein folding- and secretion-related genes. Some studies have shown that heterologous protein production upregulates the expression of ER chaperone genes, such as *bipA* (a major endoplasmic reticulum chaperone protein gene), *prpA* (a PDI [protein disulfide isomerase]-related gene), and *clxA* (a calnexin homologue from *Aspergillus niger*) (Punt et al., 1998; Wang and Ward, 2000; Wang et al., 2003). To examine the effects of carrier fusion on the UPR and secretion of the target protein, a DNA microarray was used to analyze the transcriptional level of the *A. oryzae* whole genome using CHY as a model heterologous protein (Ohno et al., 2011). The production level of a carrier-fused CHY with α-amylase increased by approximately twofold in comparison with the non-carrier-fused CHY, and several genes involved in ER protein folding and secretion were significantly upregulated in the carrier fusion CHY strain by DNA microarray analysis. Similarly, the transcripts of *hacA*, which encodes the UPR transcription factor required for upregulation of the ER chaperone and foldase-encoding genes (Mulder et al., 2006), were effectively spliced in the carrier-fused CHY strain. These results suggest that carrier-fused CHY temporarily accumulated in the ER and remarkably induced UPR in the carrier-fused strain (Ohno et al., 2011). However, recent studies have shown that highly expressed endogenous secretory proteins can also evoke the UPR. This finding raises the possibility that unfolded or misfolded proteins are selectively recognized by quality control mechanisms in filamentous fungi (Yokota et al., 2017).

Protein trafficking through the secretory pathway depends on the production levels of foldases and chaperones, which play crucial roles in protein folding during secretion. Some studies have shown that the overexpression of ER foldase- and molecular chaperone-related genes partially alleviate ER overload. It has been reported that the overexpression of chaperone BipA and PDI genes improve the production of heterologous proteins (Moralejo et al., 2001; Lombrana et al., 2004). Among them, the overexpression of *bipA* increased thaumatin secretion by 2- to 2.5-fold compared to the control strain; conversely, homologous protein secretion was not influenced by its overexpression in *Aspergillus awamori*.

In addition, vacuolar protein sorting (VPS), autophagy, and ER-Golgi cargo receptors also affect the heterologous protein production of *A. oryzae* (Yoon et al., 2013; Hoang et al., 2015). Autophagy is a highly conserved intracellular degradation pathway in eukaryotes that can recycle intracellular components as a survival mechanism under nutritional starvation (Reggiori and Klionsky, 2002). Furthermore, autophagy was confirmed to deliver misfolded secreted proteins accumulated in the ER to vacuoles, which is an important process that adversely affects heterologous protein production in *A. oryzae* (Kimura et al., 2011). In a previous study, according to the homology analysis with *S. cerevisiae*, five putative autophagy-related genes: *Aoatg1*, encoding a kinase involved in autophagy induction; *Aoatg4* and *Aoatg8*, which are essential genes for the formation of autophagosomes and membrane fusion; *Aoatg13*, encoding a component of the AoAtg1 complex; and *Aoatg15*, encoding a lipase required for the breakdown of autophagic bodies, were selected and successfully deleted in *A. oryzae* (Yoon et al., 2013). Using CHY as a reporter heterologous protein, the production level of CHY in the *Aoatg1*, *Aoatg4*, and *Aoatg8* deletion strains was improved by 2.3-, 3.1-, and 2.5-fold, respectively, compared to the control strain.

In addition, homologs of several VPS genes, which play crucial roles in the secretory pathway, have been identified and characterized (Lemmon and Traub, 2000; Suzuki et al., 2003). Among them, VPS10 has been identified to encode a sorting receptor for the recognition and delivery of some vacuolar proteins in yeast (Cooper and Stevens, 1996). Although it was also found to target aberrant and recombinant proteins for vacuolar degradation, the effect of *VPS10* gene deletion on the production of heterologous proteins is unclear. Studies have shown that the *vps10* gene deletion resulted in missorting of vacuolar carboxypeptidase CpyA and was then secreted into the medium (Marcusson et al., 1994), indicating that Vps10 is necessary for sorting vacuolar proteins into vacuoles. The analysis of heterologous protein production indicated that the *Aovps10* deletion mutation elevated the maximum extracellular production levels of the heterologous proteins HLY and CHY by 2.2- and 3-fold, respectively (Yoon et al., 2010). These results revealed that AoVps10 is involved in the regulation of heterologous protein secretion and vacuolar protein degradation in *A. oryzae*.

### Promoters and Endogenous Secretion Carrier Proteins for Heterologous Gene Expression

To improve the production of heterologous proteins, in addition to reducing the activity of proteases, a series of strong promoters were developed to induce the expression of heterologous genes (Table 2). In *A. oryzae*, the starch-inducible promoter of the alpha-amylase gene (P*amyB*) is one of the most commonly used promoters for the production of foreign proteins (Tsuchiya et al., 1992; Ward et al., 1992; Rey et al., 2003; Hoshida et al., 2005; Yamashita et al., 2011; Yin et al., 2015). The glucoamylase gene promoter (P*glaA*) and its improved promoter (P*glaA142*) were often used for the production and function analysis of recombinant proteins, such as *Candida antarctica* lipase B and *A. oryzae* recombinant tannase (Tamalampudi et al., 2007; Tokuoka et al., 2008; Ichikawa et al., 2020). The constitutive *gpdA* gene promoter from *A. nidulans* (de Ruiter-Jacobs et al., 1989; te Biesebeke et al., 2006; Punya et al., 2013), thiamine-regulatable promoter of the thiamine thiazole synthase gene (P*thiA*) (Shoji et al., 2005), and the promoter of the translation-elongation factor 1 alpha gene (P*tef1*) (Kitamoto et al., 1998) were also used for homologous and heterologous protein production in *A. oryzae*. In addition, the glucoamylase-encoding gene (*glaB*) promoter was developed for recombinant protein production in solid-state fermentation of *A. oryzae* (Ishida et al., 2006), and the promoter of the oxidoreductase gene *kojA* involved in the kojic acid biosynthesis, was successfully used to induce the expression of the polyketide synthase gene (*wA*) by *A. oryzae* (Tamano et al., 2019). The promoters used for *A. oryzae* recombinant protein production are summarized in Table 2. In addition to these promoters, studies have shown that the alterations in 5′ untranslated region (5′ UTR) importantly improved the translation efficiency of heterologous proteins in *A. oryzae* (Koda et al., 2004, 2006). The results suggested that the 5′ UTR can be used in combination with various strong promoters to enhance the expression of heterologous proteins. Furthermore, the host endogenous high-secreted protein genes were also further developed for the fusion and expression of exogenous protein genes (Gasser and Mattanovich, 2007; Jin et al., 2007; Hoang et al., 2015). Current studies have shown that the fusion of exogenous protein genes into the end of the highly secreted endogenous protein genes of *A. oryzae* reduce the degradation of heterologous proteins by the protease of *A. oryzae* (Ohno et al., 2011). For example, when a reporter heterologous protein CHY was expressed with fused α-amylase (AmyB), which is one of the most abundantly expressed proteins in *A. oryzae* under starch-induced culture conditions, the production level increased by approximately twofold compared to the non-carrier fused CHY (Ohno et al., 2011). Another study showed that using the *A. oryzae* glucoamylase gene (*glaA*) as a carrier protein, carrier-fused recombinant chymosin was secreted at a high level and showed a fivefold higher chymosin production level compared with the control using the *glaA* promoter alone (Tsuchiya et al., 1994). Furthermore, a mite allergen Der f 7 gene was highly expressed by fusion with the *A. oryzae* glucoamylase carrier protein, and its expression level was further enhanced by codon optimization which is also an effective method for improving the production levels of heterologous proteins (Tokuoka et al., 2008; Tanaka et al., 2012).

**TABLE 2:** Promoters used for the production of recombinant proteins in *Aspergillus oryzae.*

Promoter gene	Species	Description	Produced recombinant protein	References
*amyB*	*Aspergillus oryzae*	An alpha-amylase gene (*amyB*) promoter.	A mature human lysozyme (HLY);	Tsuchiya et al., 1992
			Recombinant human lactoferrin	Ward et al., 1992
			*Thielavia terrestris* glucoamylase	Rey et al., 2003
			*Pycnoporus coccineus* extracellular laccase	Hoshida et al., 2005
			*Aspergillus fumigatus* elastase inhibitor AFUEI	Yamashita et al., 2011
			A novel fungal α-amylase (PcAmy) gene from *Penicillium* sp.	Yin et al., 2015
			Leucine aminopeptidase A (*lapA*) from *A. oryzae*	Matsushita-Morita et al., 2011
*glaA142*	*A. oryzae*	An improved glucoamylase gene promoter P*glaA142*	*Candida antarctica* lipase B (CALB)	Tamalampudi et al., 2007
			Mite allergen Der f 7	Tokuoka et al., 2008
			*A. oryzae* enzymes (BglA, BglF, and BglJ)	Kudo et al., 2015
			*A. oryzae* recombinant tannase (*AotanB*)	Ichikawa et al., 2020
*glaB*	*A. oryzae*	A glucoamylase-encoding gene (*glaB*) promoter for recombinant protein production in solid-state fermentation of *A. oryzae*	*A. oryzae* glucoamylase A (*glaA*) and glucoamylase B (*glaB*)	Ishida et al., 2006
*tef1*	*A. oryzae*	Promoter of a translation-elongation factor 1 alpha gene, *tef1*	Polygalacturonase (*pgaA* and *pgaB*)	Kitamoto et al., 1998
*melO*	*A. oryzae*	The tyrosinase-encoding gene (*melO*) promoter	*E. coli* beta-glucuronidase (GUS)	Ishida et al., 2001
*gpdA*	*Aspergillus nidulans*	The constitutive *gpdA* promoter from *A. nidulans*	Bacterial beta-galactosidase (*lacZ*) and beta-glucuronidase (*uidA*)	de Ruiter-Jacobs et al., 1989
			GFP and DsRed fluorescent proteins	Nguyen et al., 2016
			Seven polyketide synthase (PKS) genes from *Xylaria* sp.	Punya et al., 2013
			Hemoglobin domains (HBD) isolated from *A. oryzae* and *Aspergillus niger*	te Biesebeke et al., 2006
*thiA*	*A. oryzae*	The thiamine-regulatable promoter of the thiamine thiazole synthase gene (P*thiA*)	Enhanced green fluorescent protein (EGFP)	Shoji et al., 2005
*enoA142*	*A. oryzae*	An improved enolase promoter (P-*enoA142*)	*Fusarium heterosporum* lipase (FHL)	Takaya et al., 2011
		An improved *enoA* promoter that harbored 12 tandem repeats of the *cis*-acting element (region III) of *A. oryzae*	Beta-glucuronidase (GUS)	Tsuboi et al., 2005
*kojA*	*A. oryzae*	A promoter of the secondary metabolism gene *kojA* involved in the kojic acid biosynthesis, for the production of other secondary metabolites by *A. oryzae*	A polyketide synthase (*wA*) of *A. oryzae*	Tamano et al., 2019
*hlyA*	*A. oryzae*	A hemolysin-like protein encoding gene (*hlyA*) promoter used for protein overexpression in *A. oryzae* grown in solid-state culture	*A. oryzae* endo-1,4-β-glucanase (CelA) and endoglucanase CelB; *Trichoderma reesei* endoglucanases (TrEglI and TrEglIII)	Bando et al., 2011
*actB*	*A. oryzae*	Promoter of an actin-encoding *actB* gene that can be highly induced by benomyl treatment	Antifungal compounds	Marui et al., 2010b
*pgkA*	*A. oryzae*	A constitutive phosphoglycerate kinase (PGK) gene promoter	PEP carboxylase (PPC) or PEP carboxykinase (PCK) genes from *E. coli*	Liu et al., 2017
*sodM*	*A. oryzae*	Promoter of manganese superoxide dismutase-encoding gene *sodM*	Beta-glucuronidase (GUS)	Ishida et al., 2004
			6-phosphofructokinase (*pfk*)	Liu et al., 2017

### Applications of Other Biotechnological Research Methods

Random mutations caused by ultraviolet radiation is a traditional method to improve the production capacity of heterologous proteins in filamentous fungi. Through the UV radiation mutation method, an *A. oryzae* superproductive mutant (AUT1-8) was isolated using HLY as a screening indicator (Nemoto et al., 2009). First, HLY-hyperproducing mutants were screened from the *tppA* and *pepE* disruptants through a halo assay based on HLY activity. Then, the plasmid containing the lysozyme gene was removed from the mutants, and the resulting strains were named AUT strains. The AUT1 strain showed production levels of heterologous proteins HLY and CHY that were 2.6 and 3.2 times higher than those of the control strains, respectively (Nemoto et al., 2009). By utilizing next-generation DNA sequencing technology and comparative genomics analysis, some potential protein production-related mutation sites were identified in the *A. oryzae* mutant (Jin et al., 2016). Among these genes, an *autA* gene (AO090120000003), which was predicted to encode a cytoplasmic protein with unknown function, including an alpha/beta hydrolase fold domain, was identified for the first time to be responsible for high-level heterologous protein production in the AUT1 strain. The HLY production level of the *autA* mutant or deletion strains was twofold higher than that of the control strain, especially in the early growth stage of cultivation. To further improve heterologous protein production, using the generated hyperproducing mutant strain of *A. oryzae*, AUT1, double deletion of the *Aovps10* and *AosedD* genes mentioned above was performed, which increased the yield of HLY and CHY by 2.1- and 1.6-fold, respectively, compared to the parental strain (Zhu et al., 2013). Thus, an excellent fungal host for the production of heterologous recombinant protein was generated by combining the mutation method with molecular breeding techniques.

Through ultraviolet radiation, Murthy and Kusumoto (2015) also obtained an *A. oryzae* mutant with a high acid protease yield, which was 5.6 times higher than that of the parent strain. In addition, through exposure to different doses of gamma irradiation, two maximum kojic acid overproduction mutants were attained from *A. flavus* HAk1 and *A. oryzae* HAk2, and the yield of kojic acid of the two mutants was 1.9- and 2.03-fold higher than that of their wild-type strains under malt extract sucrose culture conditions (Ammar et al., 2017).

The *A. oryzae* solid-state fermentation has been confirmed to produce high levels of hydrolases critical to the fermentation process. The genomic comparison and transcriptomic analysis revealed that the extracellular hydrolase genes were highly induced during solid-state cultivation in *A. oryzae* (Machida et al., 2008). Studies have shown that some proteinases and glycoside hydrolases have higher production level in solid-state fermentation than in submerged fermentation by *A. oryzae* (Zhao et al., 2019; Melnichuk et al., 2020). In addition, a glucoamylase gene *glaB* expressed exclusively in solid-state culture, therefore, as mentioned above, its promoter has been used for the production of recombinant proteins in solid-state fermentation of *A. oryzae* (Hata et al., 1998; Ishida et al., 2006). Recent studies have also shown that the transcription factor FlbC is involved in the GlaB production in solid-state culture (Tanaka et al., 2016; Gomi, 2019). These advances in molecular biology are expected to be better applied to the production of homologous and heterologous production in solid-state fermentation of *A. oryzae*. A transposable element known as a DNA sequence can change its relative position within the genome. According to their different mechanisms of transposition, transposable elements can be divided into two classes: retrotransposons, which replicate through an RNA intermediate and introduce into multiple loci of the genome, and DNA transposons, which transpose directly by a ‘cut-and paste’ mechanism through a DNA form (Daboussi, 1997; Daboussi and Capy, 2003). Although the *A. oryzae* genome sequence database (Machida et al., 2005) showed that a DNA transposon and some retrotransposons have been identified, their transposition activities have not yet been detected. In recent years, an active class II DNA transposon, *crawler*, has been identified in an industrial production strain of *A. oryzae* (OSI1013) under appropriate conditions (Ogasawara et al., 2009). In addition, in a constructed chromosomally reduced mutant of *A. oryzae*, the copy number of a gene (AO090005001597), which is a part of an LTR retrotransposable element, increased significantly (Jin et al., 2014). The discovery of this phenomenon is expected to improve the production capacity of heterologous recombinant proteins by constructing multicopy exogenous protein production strains in *A. oryzae* in the future.

## Production of Secondary Metabolites in *A. oryzae*

As a versatile fermentative strain, *A. oryzae* cannot only produce a large number of amylases and proteases but also produce useful secondary metabolites; however, the research progress in this area is relatively slow. Moreover, *A. oryzae*, as the host for fungal secondary metabolite production, has a significant advantage in identifying the function of secondary metabolic genes compared to other filamentous fungi such as *A. nidulans*, in which almost no production of endogenous secondary metabolites was found. With the development of a large number of *Aspergillus* genome sequences in recent years, the production of some metabolites can be predicted through a comparative genomics approach, among which only a small part of the related gene clusters of secondary metabolites have been identified (Ehrlich and Mack, 2014; Takeda et al., 2014; Kjaerbolling et al., 2020). For example, genomic comparison analysis has shown that *A. oryzae* retains a highly intact gene cluster that produces penicillin but has very low yields. By artificially regulating the overexpression of biosynthetic genes by strong promoters, the yield of penicillin was increased by 100 times in *A. oryzae* (Marui et al., 2010a). In addition to penicillin, *A. oryzae* also produces useful secondary metabolites such as kojic acid (Marui et al., 2011; Yamada et al., 2014; Ammar et al., 2017). In addition, using the Cre/*loxP*-mediated marker recycling system, hyperproduction of kojic acid was achieved in *A. oryzae* by introducing the oxidoreductase gene *kojA* and transporter gene *kojT* (Zhang et al., 2017).

Epigenetic manipulation through the gene disruption or drug treatment [e.g., histone deacetylase inhibitors trichostatin A (TSA) and suberoylanilide hydroxamic acid (SAHA)] is one of the trend for waking up silent secondary metabolite genes cluster. It has been reported that the addition of the histone deacetylase inhibitor SAHA markedly improved the diversity of secondary metabolites in filamentous fungi (Zhu et al., 2019). In *A. oryzae*, through screening a gene-disruption library of transcription factors, Shinohara et al. (2016) found that the production of some secondary metabolites including astellolide F (14-deacetyl astellolide B) were significantly increased by the disruption of *cclA*, which encodes a component of the histone 3 lysine 4 (H3K4) methyltransferase complex of proteins associated with Set1 complex. Kawauchi et al. (2013) also showed that the fungus-specific sirtuin HstD/AoHst4 could coordinate the production of secondary metabolites by regulating the expression of *laeA*, which encodes a global regulator importantly involved in the regulation of fungal development and secondary metabolism.

Some studies have shown that *A. oryzae* can also be used as a host for heterologous expression of biosynthetic gene clusters for useful secondary metabolites, such as diterpene aphidicolin, which is a specific inhibitor of DNA polymerase α, and plant polyketide curcumin (Fujii et al., 2011; Kan et al., 2019). In particularly, a host strain with quadruple selectable markers (Jin et al., 2004b) has also been efficiently used for heterologous production of some fungal natural products (e.g., phytotoxic metabolites, basidiomycete terpenes, and plant hormones) by reconstituting these biosynthetic gene clusters in *A. oryzae* (Nagamine et al., 2019; Takino et al., 2019; Oikawa, 2020a,b). Moreover, the overexpression of *laeA* gene was also found to promote the overexpression of two clusters of heterologous biosynthetic genes from *A. nidulans*, resulting in the production of the corresponding metabolite, monacolin K or terrequinone A (Sakai et al., 2012). In summary, although some progress has been made in the study of secondary metabolites of *Aspergillus* species in recent years (Sanchez et al., 2012; Amare and Keller, 2014; He et al., 2018; Caesar et al., 2020), there are still many unknown fields awaiting further discovery.

## Conclusion

Decoding the *A. oryzae* genome sequence provides abundant and reliable genetic information for a deep understanding of its genetic background and production performance. Subsequently, the establishment and rapid development of new genetic engineering technology in *A. oryzae* opened up more ways for its breeding of production strains and the utilization of industrial production. In recent years, although the production capacity of some heterologous proteins and secondary metabolites has been improved to some extent, it is far from the expected goal.

At present, in addition to these bottlenecks in the production of heterologous proteins mentioned above, there are still some problems that need to be further overcome, such as: (1) How to ensure high activity in the enzyme extraction process; (2) In industrial production, some macromolecular substances, such as wheat bran, rice bran, silage, corn, and soybean meal are difficult to utilize by *A. oryzae*. Therefore, biotechnological improvements are being made to degrade and utilize these substrates, thereby reducing production costs and pollution caused by raw material waste; (3) The large amount of heterologous protein production has a certain adverse effect on *A. oryzae* itself. (4) In the late growth stage of *A. oryzae*, the introduction of some strong promoters, such as *amyB* and *glaA* promoters, will be inhibited by high-concentration products, such as glucose. A study has shown that deletion of single and double *creA*/*creB* genes, which encode regulation factors involved in carbon catabolite repression, significantly improved amylase activity in submerged cultures containing high concentrations of inducing sugars compared to the control strain (Ichinose et al., 2014).

In addition, although the whole genome sequence has been deciphered, the function of most genes in *A. oryzae* is still unclear, and there remains many restrictions on its production and utilization. Therefore, there are still a large number of unknown functional genes and fields that need to be discovered and explored. Recent advances in omics techniques, such as comparative transcriptomics, proteomics and metabolomics, have opened up a new perspective for future research on functional genes and the exploration of protein secretion pathways. In summary, there remains a great space for further research and exploration on combining genetic engineering technology with *A. oryzae* production and application to obtain the breeding of highly efficient production strains.

## Author Contributions

F-JJ wrote the main manuscript text. SH and B-TW contributed to certain sections. LJ contributed to overall editing and proofreading. All the authors approved the submitted version.

## Conflict of Interest

The authors declare that the research was conducted in the absence of any commercial or financial relationships that could be construed as a potential conflict of interest.
